# Clinical and metabolic factors associated with response to postoperative radioactive iodine therapy in Chinese obese patients with papillary thyroid carcinoma

**DOI:** 10.3389/fendo.2026.1836784

**Published:** 2026-05-19

**Authors:** Yan Zhou, Yuyue Hou, Shengqing Hu, Chenxi Zhu, Mingjie Zhang, Yuangu Gao, Yajing Zhang, Ji Li, Wei Cao, Zairong Gao, Jie Tan, Xiaotian Xia

**Affiliations:** 1 Department of Nuclear Medicine, Union Hospital, Tongji Medical College, Huazhong University of Science and Technology, Wuhan, China; 2 Hubei Province Key Laboratory of Molecular Imaging, Wuhan, China; 3 Key Laboratory of Biological Targeted Therapy, the Ministry of Education, Wuhan, China; 4 Tongji Medical College, Huazhong University of Science and Technology, Wuhan, China; 5 Department of Breast and Thyroid Surgery, Union Hospital, Tongji Medical College, Huazhong University of Science and Technology, Wuhan, China

**Keywords:** high-density lipoprotein, obesity, papillary thyroid carcinoma, radioactive iodine therapy, stimulated thyroglobulin, treatment response

## Abstract

**Background and objective:**

Obesity is a heterogeneous metabolic condition that may influence tumor biology and therapeutic outcomes. However, factors associated with the response to postoperative radioactive iodine (^131^I) therapy in obese patients with papillary thyroid carcinoma (PTC) remain insufficiently understood. This study aimed to identify clinical and metabolic factors associated with treatment response in this population.

**Methods:**

This retrospective observational study included obese patients with PTC who underwent postoperative ^131^I therapy between January 2020 and December 2023. Clinical, biochemical, imaging, treatment, and follow-up data were collected from electronic medical records. Treatment response at the most recent follow-up was classified as excellent response (ER) or non-excellent response (NER) according to the 2025 American Thyroid Association (ATA) guidelines. Logistic regression analysis was performed to identify factors associated with treatment response, and receiver operating characteristic (ROC) analysis was used to evaluate predictive performance.

**Results:**

A total of 219 obese patients with PTC were included, with a median follow-up of 31.00 months. At the last follow-up, 91 patients (41.6%) achieved ER. In multivariable analysis, higher high-density lipoprotein (HDL) levels were independently associated with an increased likelihood of ER (OR = 22.891, 95% CI: 2.644-198.206, P = 0.004), whereas higher pre-ablation stimulated thyroglobulin (sTg) levels were independently associated with a lower likelihood of ER (OR = 0.793, 95% CI: 0.719-0.876, P < 0.001). Triglycerides (TG) also showed a weaker association with treatment response (OR = 0.662, 95% CI: 0.442-0.991, P = 0.045). Among the individual indicators, sTg showed the best discriminatory performance (AUC = 0.767), followed by HDL (AUC = 0.672) and TG (AUC = 0.624). A combined model incorporating HDL and sTg improved predictive performance (AUC = 0.822).

**Conclusion:**

In obese patients with PTC who underwent postoperative ^131^I therapy, pre-ablation sTg and HDL were independently associated with treatment response, whereas TG showed a weaker association. The combination of HDL and sTg provided better discriminatory performance than either marker alone. These findings suggest that combining metabolic indicators with conventional tumor-related markers may improve individualized evaluation of treatment response in obese patients with PTC.

## 1 Introduction

Thyroid cancer (TC) has exhibited the fastest increase in age-standardized incidence among all malignancies in China, with annual growth rates exceeding 7% (1). This persistent rise highlights the increasing disease burden of TC. Similar trends have been observed in many other countries and regions and are largely driven by the growing incidence of papillary thyroid carcinoma (PTC) (2). Improved detection and overdiagnosis are widely considered major contributors to this trend (3, 4). However, accumulating evidence suggests that changes in lifestyle and metabolic health may also play an important role (5, 6). Among these factors, obesity has emerged as a potential contributor to the rising incidence of PTC (7, 8). Notably, the prevalence of obesity has increased rapidly in China over recent decades, raising concerns about its possible contribution to the growing burden of TC (9, 10).

Obesity is characterized by chronic low-grade inflammation, insulin resistance, hormonal dysregulation, and altered adipokine secretion (11, 12). In obese adipose tissue, macrophage infiltration and the increased release of inflammatory cytokines and adipokines create a pro-inflammatory and metabolically dysregulated microenvironment that may promote tumor initiation and progression and potentially affect responses to cancer therapy (12–14). Consistent with these biological mechanisms, epidemiological studies have shown that obesity is associated with an increased risk of PTC (15, 16). Moreover, obese patients are more likely to present with adverse clinicopathological features, including larger tumor size, lymph node metastasis, and extrathyroidal extension (17–20). These findings suggest that obesity may not only increase the risk of developing PTC but may also influence tumor behavior and clinical outcomes.

Importantly, obesity is increasingly recognized as a heterogeneous clinical and metabolic condition (21). Even among individuals with similar body mass index (BMI), substantial differences may exist in metabolic profiles and systemic inflammatory status. In addition, clinicopathological characteristics and disease burden may vary considerably even within the obese PTC population (17). Such intragroup heterogeneity may contribute to differences in tumor behavior and clinical outcomes. Therefore, focusing specifically on obese patients may help to better characterize interindividual variability in metabolic and tumor-related factors within this clinically relevant subgroup.

Radioactive iodine (^131^I) therapy plays a central role in the postoperative management of PTC, particularly in patients at intermediate or high risk of recurrence (22–24). It is widely used to ablate remnant thyroid tissue and to treat microscopic or clinically evident metastatic disease after thyroidectomy. The therapeutic efficacy of ^131^I is influenced by multiple factors, including tumor burden, iodine avidity, and patient-specific physiological characteristics. In obese patients with PTC, heterogeneity in metabolic status, clinicopathological features, and disease burden may also contribute to variability in response to postoperative ^131^I therapy. However, the determinants of therapeutic response in this specific population remain insufficiently understood.

Therefore, the present study aimed to evaluate the therapeutic efficacy of ^131^I therapy in obese patients with PTC and to identify clinical, biochemical, imaging, and treatment-related factors associated with treatment outcomes in this specific population.

## 2 Patients and methods

### 2.1 Study design and population

This retrospective observational study included obese patients with PTC who underwent postoperative ^131^I therapy at Union Hospital, Tongji Medical College, Huazhong University of Science and Technology, between January 2020 and December 2023. Relevant clinical data were retrieved from the electronic medical record system. The study was approved by the Institutional Review Board (Approval No. UHCT250789), and the requirement for informed consent was waived because of the retrospective nature of the study.

**Figure 1:**
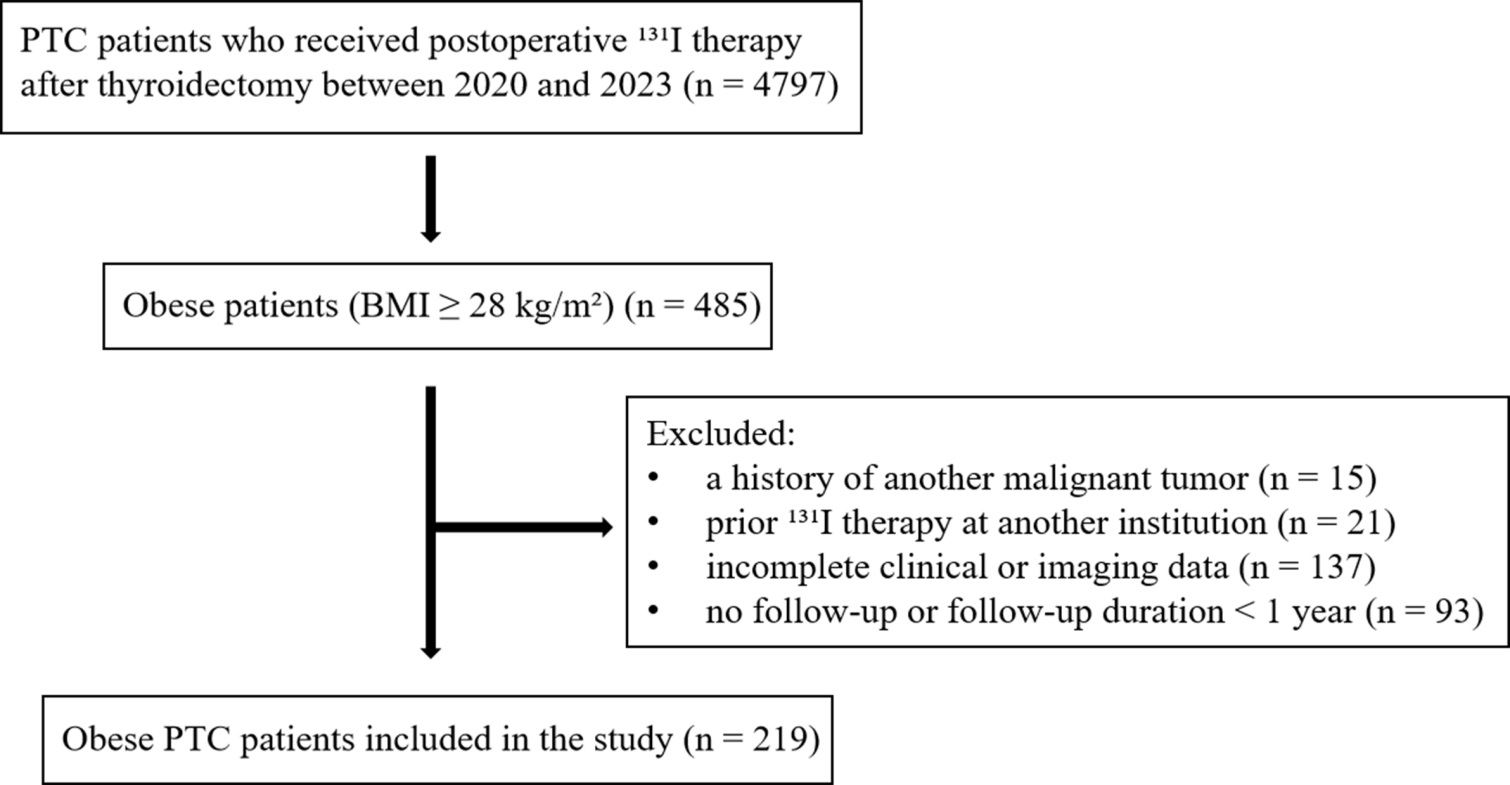
Flow chart for patient selection.

Eligible patients were screened according to the predefined inclusion and exclusion criteria, and the patient selection process is shown in **Figure 1**.

Patients were eligible for inclusion if they met all of the following criteria:

1. age ≥18 years;
2. total or near-total thyroidectomy with postoperative pathological confirmation of PTC;
3. BMI ≥28 kg/m², according to the Chinese criteria for adult obesity (10), calculated from height and weight measured at hospital admission for the initial ^131^I therapy.

Patients were excluded if any of the following conditions were present:

1. a history of another malignant tumor;

2. prior ^131^I therapy performed at another institution before the index treatment;

3. incomplete clinical or imaging data;

4. no follow-up or a follow-up duration of <1 year.

### 2.2 Diagnosis and clinicopathological classification

The diagnosis of PTC was confirmed by postoperative histopathological examination. Tumor staging was performed according to the 8th edition of the American Joint Committee on Cancer (AJCC) TNM staging system, and recurrence risk stratification was assessed according to the 2025 American Thyroid Association (ATA) guidelines (25, 26). Clinicopathological parameters used for staging and risk stratification included tumor size, tumor multifocality, extrathyroidal extension, lymph node involvement, and the presence of distant metastasis.

Additional clinicopathological variables were defined based on postoperative pathological reports. Tumor multifocality was classified as unifocal (a single tumor focus) or multifocal (two or more tumor foci) according to the number of tumor foci. Tumor size was defined as the maximum diameter of the largest tumor focus. Extrathyroidal extension, extranodal extension, the number of metastatic lymph nodes, lymph node ratio, and the maximum diameter of the largest metastatic lymph node were also recorded. The lymph node ratio was defined as the number of metastatic lymph nodes divided by the total number of dissected lymph nodes.

### 2.3 ^131^I therapy and follow-up

Patients underwent standard preparation for ^131^I therapy according to institutional protocols and relevant clinical guidelines, including thyroid hormone withdrawal (THW) and adherence to a low-iodine diet before treatment.

**Figure 2:**
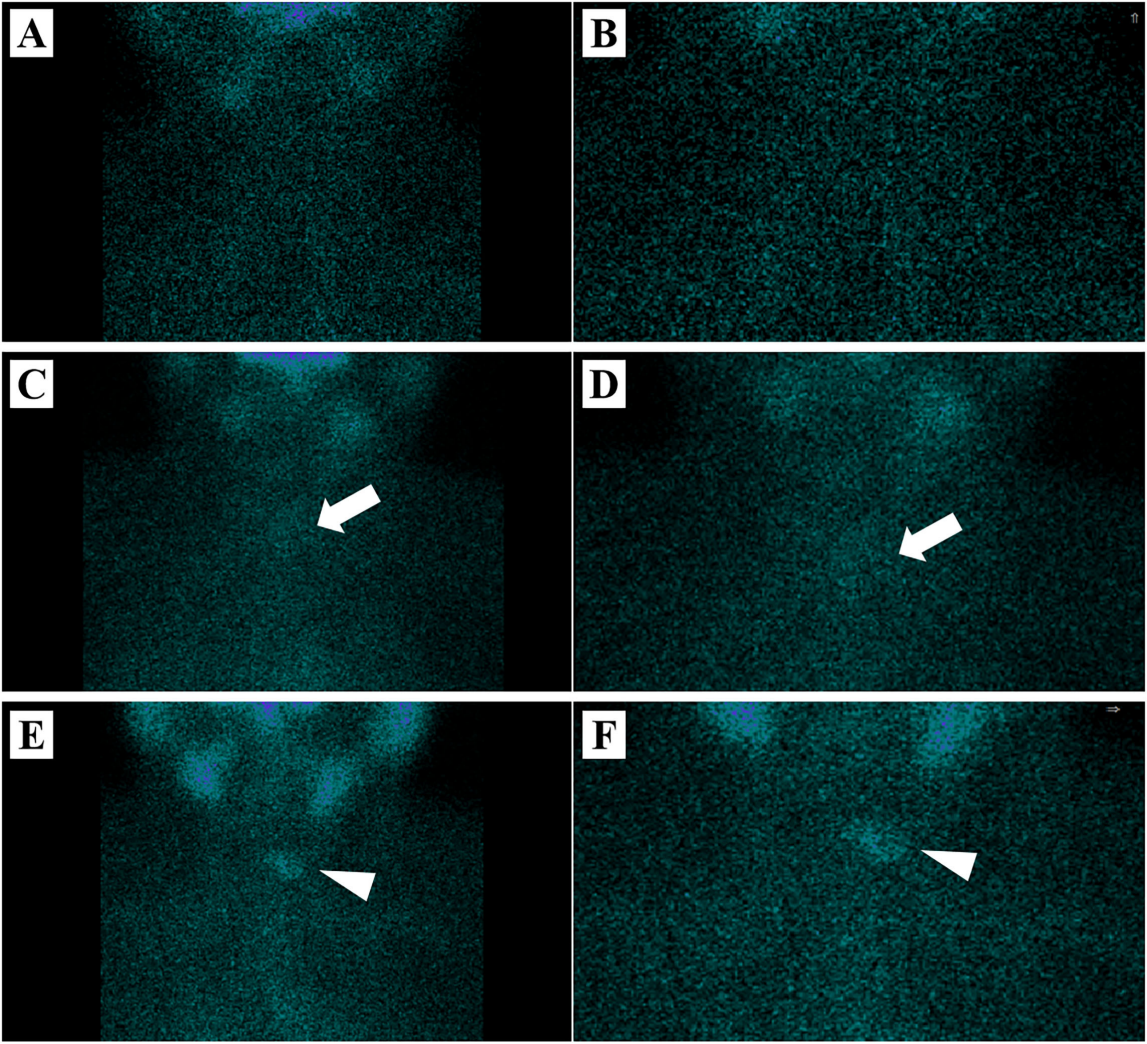
Representative thyroid images for the assessment of residual thyroid tissue. **(A, B)** None: no visible uptake in the thyroid bed. **(C, D)** Minimal: faint uptake in the thyroid region (white arrows). **(E, F)** Residual: clear and substantial uptake in the thyroid region (white triangles). The images in the right column **(B, D, F)** are 1.5-fold magnified views of the corresponding images in the left column **(A, C, E)**, respectively.

Pre-therapy assessments included complete blood count, liver and renal function tests, lipid profile, including low-density lipoprotein (LDL), high-density lipoprotein (HDL), triglycerides (TG), and total cholesterol (TC), thyroid function tests, stimulated thyroglobulin (sTg) and thyroglobulin antibody (TgAb) levels, cervical and thyroid ultrasonography, and chest CT. TgAb-positive patients were not excluded, and TgAb levels were retained as a variable in the analysis. All patients also underwent pre-therapy thyroid imaging using ^99m^Tc-pertechnetate. The images were independently reviewed by two experienced nuclear medicine physicians to evaluate residual thyroid tissue. Residual thyroid tissue was visually classified according to the presence and intensity of radiotracer uptake in the thyroid bed as none (no visible uptake), minimal (faint uptake), or residual (clear and substantial uptake). Disagreements were resolved by consensus. Representative images of each category are shown in **Figure 2** to illustrate the visual classification criteria.

The administered ^131^I activity was determined by the treating physician on the basis of ATA risk stratification and institutional practice, with reference to the 2015 ATA guidelines and relevant Chinese guidelines in effect during the study period (27–29). In general, a risk-adapted strategy was used, with lower activities for remnant ablation in lower-risk patients and higher activities in patients with greater disease burden or suspected residual or metastatic disease. This approach reflected routine clinical decision-making in our institution during the study period.

Patients underwent regular follow-up after therapy. Follow-up assessments included nonstimulated thyroglobulin (nsTg), sTg, TgAb levels, and thyroid function tests, as well as cervical ultrasonography and chest CT when clinically indicated. Follow-up duration was calculated from the date of the initial ^131^I therapy to the date of the last recorded clinical visit.

### 2.4 Evaluation of treatment response

Treatment response was evaluated on the basis of clinical, biochemical, and imaging findings at the most recent follow-up. The assessment was performed according to the Chinese Guidelines for the Diagnosis and Management of Thyroid Nodules and Differentiated Thyroid Cancer (Second Edition) and the 2025 ATA guidelines (26, 28). Therapeutic response was categorized into four groups: excellent response (ER), indeterminate response (IDR), biochemically incomplete response (BIR), and structurally incomplete response (SIR).

ER was defined as nsTg <0.2 ng/mL or sTg <1 ng/mL with negative imaging findings. IDR was defined as nonspecific findings on imaging studies, or nsTg of 0.2–1 ng/mL, or sTg of 1–10 ng/mL, or stable/declining TgAb levels. BIR was defined as nsTg >1 ng/mL or sTg >10 ng/mL, or increasing TgAb levels in the absence of structural evidence of disease on imaging. SIR was defined as structural evidence of disease, including suspicious imaging findings or biopsy-proven local or distant metastatic disease.

For regression analyses, treatment outcomes were dichotomized as ER and non-excellent response (NER), with IDR, BIR, and SIR classified as NER.

### 2.5 Statistical analysis

Continuous variables were expressed as median with interquartile range (IQR), and categorical variables were presented as counts and percentages. Comparisons between the ER and NER groups were performed to explore factors associated with treatment outcomes. Univariate logistic regression analysis was conducted to evaluate the association between clinical variables and therapeutic response to ^131^I therapy. Variables with P <0.05 in the univariate analysis were subsequently included in a multivariable logistic regression model to identify independent predictors of treatment response. Odds ratios (ORs) and 95% confidence intervals (CIs) were calculated.

Receiver operating characteristic (ROC) curve analysis was performed to assess the predictive performance of significant variables and of a combined predictive model constructed using logistic regression. The predicted probabilities derived from the logistic regression model were used to generate the ROC curve. The area under the curve (AUC), optimal cutoff value, sensitivity, and specificity were calculated.

To further evaluate the relationship between the initial ^131^I dose and therapeutic response, subgroup analyses were conducted according to ATA risk stratification. The initial ^131^I dose was modeled as a continuous variable and scaled per 10 mCi increase to facilitate clinical interpretation of the effect size, and the corresponding ORs were calculated.

All statistical analyses were performed using R software (version 4.4.0; R Foundation for Statistical Computing, Vienna, Austria). A two-sided P <0.05 was considered statistically significant.

## 3 Results

### 3.1 Patient characteristics

A total of 219 obese patients with PTC who underwent postoperative ^131^I therapy were included in the final analysis. The baseline demographic, clinicopathological, treatment, and follow-up characteristics are summarized in **Table 1**. The study population had a median age of 38 years and a median BMI of 29.54 kg/m². Most patients presented with intermediate-high risk disease features. The median tumor diameter was 1.30 cm, extrathyroidal extension was present in 155 patients (70.8%), and the median number of metastatic lymph nodes was 8. According to ATA risk stratification, the largest proportion of patients were classified as intermediate-high risk (123 patients, 56.2%), followed by high risk (60 patients, 27.4%).

**Table 1:** Baseline characteristics.

Variable	Value
Total	219
Demographic characteristics	
Sex (male/female)	130/89
Age (years)*	38 (32-45)
Residence (urban/rural)	190/29
Clinical and biochemical characteristics	
BMI (kg/m²)*	29.54 (28.44-31.22)
LDL (mmol/L)*	2.85 (2.34-3.42)
HDL (mmol/L)*	1.02 (0.90-1.21)
TG (mmol/L)*	1.66 (1.23-2.54)
TC (mmol/L)*	4.73 (4.14-5.30)
sTg (ng/mL)*	2.35 (1.56-3.33)
TgAb (IU/mL)*	3.56 (0.90-11.70)
Tumor and staging characteristics	
Tumor laterality (unilateral/bilateral)	98/121
Maximum tumor diameter (cm)*	1.30 (0.90-2.00)
Multifocality (multifocal/unifocal)	71/148
Extrathyroidal extension (yes/no)	155/64
Number of metastatic lymph nodes*	8 (4-12)
Lymph node ratio*	0.42 (0.29-0.62)
Maximum metastatic lymph node diameter (cm)*	0.50 (0.24-0.80)
Extranodal extension (yes/no)	35/184
T stage (T1/T2/T3/T4)	167/30/18/4
N stage (N0/N1a/N1b)	4/133/82
M stage (M0/M1)	218/1
Imaging and ATA risk stratification	
Thyroid imaging (none/minimal/residual)	78/34/107
ATA risk stratification (low/low-intermediate/intermediate-high/high risk)	18/18/123/60
Treatment and follow-up.	
Cumulative ^131^I dose (mCi)*	150.00 (130.00-200.00)
Number of ^131^I treatments (1/2/3)	166/51/2
Follow-up duration (months)*	31.00 (22.00-43.50)
Treatment response (ER/NER)	91/128

*Data are presented as counts or median (IQR).

The median administered ^131^I activity was 150.00 mCi (IQR: 130.00-200.00 mCi), and the median follow-up duration was 31.00 months (IQR: 22.00-43.50 months). At the last follow-up, 91 patients (41.6%) achieved an ER, whereas 128 patients (58.4%) were classified as having a NER.

### 3.2 Univariate analysis of factors associated with therapeutic response

Univariate logistic regression analysis was performed to identify factors associated with therapeutic response to ^131^I therapy in obese patients with PTC. The results are presented in **Table 2**.

**Table 2:** Univariate logistic regression analysis of factors associated with therapeutic response to ^131^I therapy.

Variable	OR	95% CI	P value
Demographic characteristics			
Sex (male vs female)	0.626	0.361-1.082	0.094
Age (years)	1.022	0.992-1.053	0.162
Residence (urban vs rural)	1.008	0.459-2.275	0.984
Clinical and biochemical characteristics			
BMI (kg/m²)	0.891	0.793-0.992	0.042
LDL (mmol/L)	1.119	0.811-1.549	0.494
HDL (mmol/L)	6.389	2.137-21.397	0.002
TG (mmol/L)	0.663	0.498-0.846	0.002
TC (mmol/L)	1.189	0.890-1.598	0.244
sTg (ng/mL)	0.863	0.803-0.915	<0.001
TgAb (IU/mL)	1.000	0.999-1.001	0.781
Tumor and staging characteristics			
Tumor laterality (bilateral vs unilateral)	0.907	0.528-1.559	0.724
Maximum tumor diameter (cm)	0.715	0.528-0.928	0.019
Multifocality (multifocal vs unifocal)	0.958	0.541-1.708	0.884
Extrathyroidal extension (yes vs no)	0.672	0.373-1.211	0.185
Number of metastatic lymph nodes	0.942	0.897-0.987	0.015
Lymph node ratio	0.214	0.070-0.592	0.004
Maximum metastatic lymph node diameter (cm)	0.414	0.205-0.735	0.007
Extranodal extension (yes vs no)	0.431	0.182-0.939	0.042
T stage (T2 vs T1)	0.613	0.261-1.362	0.242
T stage (T3 vs T1)	0.350	0.096-1.024	0.075
T stage (T4 vs T1)	1.227	0.144-10.420	0.840
N stage (N1a vs N0)	0.250	0.012-2.010	0.235
N stage (N1b vs N0)	0.203	0.010-1.661	0.175
Imaging and ATA risk stratification			
Thyroid imaging (minimal uptake vs none)	1.135	0.499-2.559	0.761
Thyroid imaging (residual uptake vs none)	1.004	0.555-1.822	0.990
ATA risk stratification (low-intermediate risk vs low risk)	0.800	0.211-2.978	0.739
ATA risk stratification (intermediate-high risk vs low risk)	0.714	0.257-1.929	0.507
ATA risk stratification (high risk vs low risk)	0.243	0.078-0.728	0.012

M stage was excluded from regression analysis because only one patient had M1 disease. Cumulative ^131^I dose and number of treatment sessions were not included in the regression models because they were determined by disease status and subsequent clinical course, and their inclusion could introduce reverse causation bias.

Among the metabolic variables, HDL was significantly associated with therapeutic response (OR = 6.389, 95% CI: 2.137-21.397, P = 0.002), indicating a positive association with treatment efficacy. By contrast, higher TG levels (OR = 0.663, 95% CI: 0.498-0.846, P = 0.002) and higher BMI (OR = 0.891, 95% CI: 0.793-0.992, P = 0.042) were negatively associated with achieving ER.

Lymph node-related parameters, including the number of metastatic lymph nodes (OR = 0.942, 95% CI: 0.897-0.987, P = 0.015), lymph node ratio (OR = 0.214, 95% CI: 0.070-0.592, P = 0.004), and the maximum diameter of metastatic lymph nodes (OR = 0.414, 95% CI: 0.205-0.735, P = 0.007), were also significantly associated with treatment outcomes.

Among the biochemical markers, higher sTg levels were significantly associated with a lower likelihood of achieving ER (OR = 0.863, 95% CI: 0.803-0.915, P <0.001).

Other variables, including sex, age, residence, LDL, TC, tumor laterality, multifocality, extrathyroidal extension, T stage, N stage, TgAb level, and thyroid imaging findings, were not significantly associated with treatment efficacy (all P >0.05).

### 3.3 Multivariable analysis and ROC analysis

Variables with P <0.05 in the univariate analysis were entered into the multivariable logistic regression model to identify independent factors associated with therapeutic response to ^131^I therapy.

The multivariable analysis showed that HDL level and pre-ablation sTg were independently associated with treatment response. Higher HDL levels were significantly associated with an increased likelihood of achieving ER (OR = 22.891, 95% CI: 2.644-198.206, P = 0.004). In contrast, higher sTg levels were independently associated with a lower probability of treatment success (OR = 0.793, 95% CI: 0.719-0.876, P <0.001). In addition, TG remained significantly associated with therapeutic response, although the effect size was relatively modest (OR = 0.662, 95% CI: 0.442-0.991, P = 0.045).

Other variables, including BMI, maximum tumor diameter, extranodal extension, ATA risk stratification, number of metastatic lymph nodes, lymph node ratio, and maximum metastatic lymph node diameter, were not independently associated with treatment response (all P >0.05). Detailed results are shown in **Table 3**.

**Table 3:** Multivariate logistic regression analysis of factors associated with therapeutic response to ^131^I therapy.

Variable	OR	95% CI	P value
BMI (kg/m²)	0.868	0.728-1.035	0.114
HDL (mmol/L)	22.891	2.644-198.206	0.004
TG (mmol/L)	0.662	0.442-0.991	0.045
sTg (ng/mL)	0.793	0.719-0.876	<0.001
Maximum tumor diameter (cm)	0.850	0.598-1.207	0.363
Number of metastatic lymph nodes	0.927	0.837-1.026	0.142
Lymph node ratio	1.385	0.238-8.065	0.717
Maximum metastatic lymph node diameter (cm)	0.801	0.299-2.146	0.659
Extranodal extension (yes vs no)	1.313	0.123-13.965	0.821
ATA risk stratification (low-intermediate risk vs low risk)	0.256	0.031-2.103	0.205
ATA risk stratification (intermediate-high risk vs low risk)	0.681	0.102-4.540	0.691
ATA risk stratification (high risk vs low risk)	0.449	0.026-7.784	0.582

**Figure 3:**
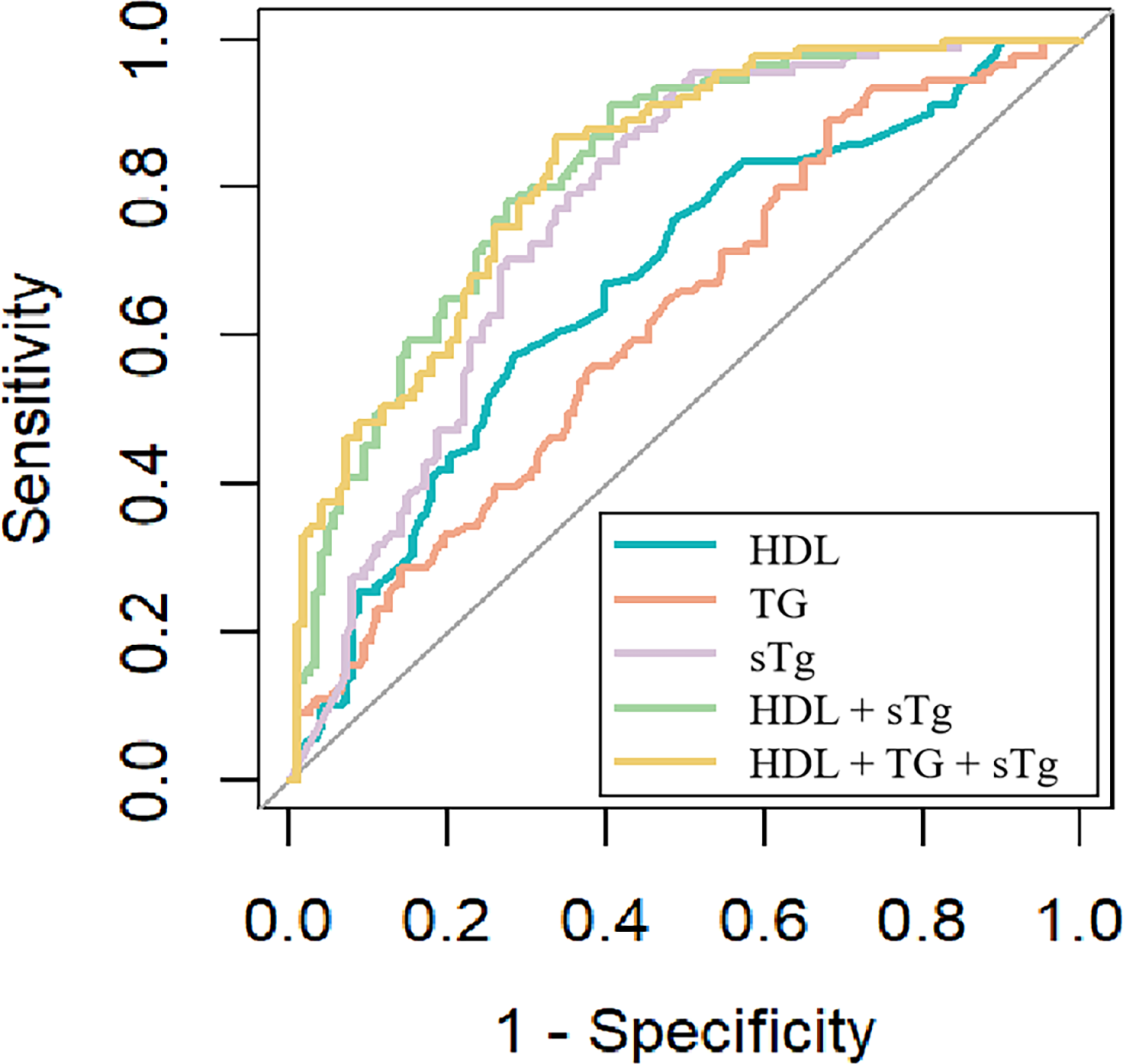
ROC curves of HDL, TG, sTg, and the combined model for predicting response to ^131^I therapy.

ROC curve analysis was performed to assess the predictive performance of HDL, TG, and sTg for therapeutic response to ^131^I therapy. Among the individual indicators, sTg showed the best discriminatory performance (AUC = 0.767, 95% CI: 0.705-0.829), followed by HDL (AUC = 0.672) and TG (AUC = 0.624) (**Figure 3**).

A combined logistic regression model incorporating HDL and sTg further improved predictive performance (AUC = 0.822). The addition of TG to the model resulted in only a marginal increase in the AUC (0.825), suggesting limited incremental predictive value. Detailed ROC results are presented in **Table 4**.

**Table 4:** Individual and combined biomarkers for predicting response to ^131^I therapy.

Model	AUC (95%CI)	Cutoff	Sensitivity	Specificity
HDL	0.672 (0.600-0.744)	1.075	0.571	0.719
TG	0.624 (0.550-0.699)	2.595	0.890	0.320
sTg	0.767 (0.705-0.829)	7.515	0.934	0.516
HDL + sTg	0.822 (0.767-0.876)	0.478	0.780	0.727
HDL + TG + sTg	0.825 (0.771-0.878)	0.407	0.868	0.664

### 3.4 Association between the initial ^131^I dose and treatment response across recurrence risk categories

We further evaluated the association between the initial ^131^I dose and therapeutic response across ATA risk categories. A significant positive association between the initial ^131^I dose and treatment response was observed only in the intermediate-high risk group, in which each 10 mCi increase in the initial dose was associated with a 37.4% increase in the odds of achieving ER (OR = 1.374, 95% CI: 1.117-1.691, P = 0.003).

No statistically significant associations were observed in the low risk, low-intermediate risk, or high risk groups. Detailed results are presented in **Table 5**.

**Table 5:** Association between initial ^131^I dose and therapeutic response across ATA risk stratification.

ATA risk stratification	OR (per 10 mCi increase)	95% CI	P value
Low risk	1.206	0.984-1.479	0.071
Low-intermediate risk	1.185	0.868-1.617	0.284
Intermediate-high risk	1.374	1.117-1.691	0.003
High risk	1.159	0.971-1.382	0.101

ORs represent the change in the odds of achieving an ER for each 10 mCi increase in the initial ^131^I dose.

## 4 Discussion

In this retrospective study of obese patients with PTC who underwent postoperative ^131^I therapy, we found that pre-ablation sTg and HDL were independently associated with treatment response, whereas TG showed a weaker association. In addition, the combination of HDL and sTg demonstrated better discriminatory performance than either marker alone. These findings suggest that both pre-treatment tumor-related and metabolic indicators may be relevant when evaluating response to postoperative ^131^I therapy in this specific patient population.

Obesity is increasingly recognized as a heterogeneous metabolic condition that may influence tumor biology and therapeutic outcomes. Chronic inflammation, insulin resistance, and altered adipokine signaling have been proposed as potential mechanisms linking obesity to cancer progression and treatment response (11–14). In obese patients with PTC, such metabolic alterations may contribute to interindividual variability in the effectiveness of postoperative ^131^I therapy. However, the present study was not designed to determine the mechanistic relationship between metabolic abnormalities and tumor burden, and our findings should therefore be interpreted primarily as clinical associations.

In the present study, pre-ablation sTg was independently associated with treatment response. Higher sTg levels were associated with a lower probability of achieving an ER after therapy. Clinically, elevated pre-ablation sTg is generally considered to reflect a greater burden of residual thyroid tissue and/or persistent disease (30). This interpretation is consistent with previous studies showing that sTg is an important biomarker for predicting treatment response and disease status in differentiated TC (30, 31). In our ROC analysis, sTg showed the highest discriminatory performance among the individual indicators, further supporting its clinical value in the evaluation of postoperative treatment outcomes.

Among the metabolic variables examined in this study, HDL emerged as an independent factor positively associated with treatment response. HDL is well known for its anti-inflammatory, antioxidative, and endothelial-protective properties, and reduced HDL levels are commonly associated with metabolic syndrome and systemic inflammation (32). In line with these biological characteristics, recent evidence has shown that low HDL levels are independently associated with an increased risk of recurrence in patients with PTC (33). In our cohort, higher HDL levels were associated with a greater likelihood of achieving an ER after ^131^I therapy, suggesting that HDL may serve as a clinically relevant marker of host metabolic status in obese patients with PTC. Higher HDL levels may reflect a less inflammatory and less oxidative metabolic state, which could be more favorable for treatment response. Given the potential role of oxidative stress in thyroid cancer progression, dedifferentiation, and regulation of sodium-iodide symporter expression and function, this may partly explain the association between higher HDL levels and a better response to postoperative ^131^I therapy (34, 35). However, this interpretation remains speculative, and the confidence interval was relatively wide; therefore, the robustness of this association should be interpreted with caution and requires validation in larger cohorts.

TG also showed a modest but statistically significant association with treatment response. Elevated TG levels are frequently linked to insulin resistance and metabolic dysregulation in obese individuals and may reflect an unfavorable systemic metabolic state (20, 36). However, compared with sTg and HDL, the magnitude and incremental value of this association were limited. Therefore, TG may be better interpreted as a secondary metabolic correlate rather than a major determinant of treatment response in the present study.

Notably, the combination of HDL and sTg provided better discriminatory performance than either indicator alone. This finding suggests that metabolic status and tumor-related markers may offer complementary information for predicting therapeutic response. However, our data do not establish a direct biological or causal link between these two domains. Rather, they indicate that combining these variables may improve clinical stratification in obese patients with PTC undergoing postoperative ^131^I therapy.

We also explored the association between the initial ^131^I dose and treatment response across ATA risk categories. A significant positive association was observed only in the intermediate-high risk group. Because the administered dose is closely linked to baseline recurrence risk and clinical decision-making, this analysis should be considered exploratory. The observed subgroup-specific association may reflect differences in disease severity, sample distribution, or treatment selection, and should therefore be interpreted cautiously rather than as a definitive dose-response conclusion.

This study has several limitations. First, it was a retrospective single-center study, which may have introduced selection bias and limited the generalizability of the findings. Second, the sample size, particularly in some subgroup analyses, may have reduced statistical power. Third, although this study focused on obese patients, the metabolic indicators analyzed were mainly lipid parameters and therefore did not fully capture the complexity of obesity. Dyslipidemia does not necessarily equate to obesity-related metabolic dysfunction, and additional anthropometric or metabolic indicators, such as waist circumference, waist-to-hip ratio, body composition measures, or indices of insulin resistance, may provide a more comprehensive assessment of obesity-related metabolic status. Waist circumference was not included in the present analysis because these data were not consistently available in the retrospective medical records.

In addition, the relatively high proportion of male patients in our cohort differs from the usual female predominance of PTC. This pattern likely reflects the restriction of the study population to obese individuals rather than the sex distribution of PTC in the general population; similar sex distributions have been reported in previous studies of obese patients with differentiated thyroid carcinoma (37). Because males generally tend to have lower HDL and higher TG levels (38), this imbalance may have influenced the distribution of metabolic parameters and, consequently, the observed associations. Furthermore, both BMI and lipid profiles were assessed at the time of admission for initial ^131^I therapy after routine THW. THW-induced hypothyroidism may lead to short-term weight changes and transient increases in TC, LDL, and TG levels (39, 40). Although all patients underwent a similar preparation protocol, these effects may have influenced baseline metabolic measurements and their associations with treatment response. TgAb positivity may also interfere with the interpretation of sTg. In the present study, TgAb-positive patients were retained to preserve real-world clinical characteristics, and TgAb was included as a variable in the analysis; however, residual confounding related to TgAb interference cannot be completely excluded. In addition, residual thyroid tissue was assessed using qualitative visual interpretation of ^99m^Tc-pertechnetate images, which may be subject to interobserver variability despite independent review by two experienced nuclear medicine physicians and resolution by consensus. Finally, treatment response was assessed at the most recent follow-up, and variation in follow-up duration across patients may have introduced heterogeneity in response classification.

Moreover, because the study focused exclusively on obese patients, no non-obese comparison group was available. Therefore, the present findings should be interpreted as reflecting variability within the obese population rather than as direct evidence of differences between obese and non-obese patients. More detailed BMI-based subgroup analyses were also not performed because they would have substantially reduced subgroup sample sizes and statistical power.

These limitations should be considered when interpreting the findings, and external validation in larger prospective and multicenter cohorts is warranted.

Taken together, our findings suggest that pre-ablation sTg and HDL are associated with treatment response in obese patients with PTC who undergo postoperative ^131^I therapy. These results may provide useful information for treatment evaluation in this specific population, although further prospective and multicenter studies are needed to confirm their clinical applicability.

## 5 Conclusion

In obese patients with PTC who underwent postoperative ^131^I therapy, pre-ablation sTg and HDL were independently associated with treatment response, whereas TG showed a weaker association. The combination of HDL and sTg provided better discriminatory performance than either marker alone. These findings suggest that combining metabolic indicators with conventional tumor-related markers may improve individualized evaluation of treatment response in obese patients with PTC.

## Data Availability

The raw data supporting the conclusions of this article will be made available by the authors, without undue reservation.
